# 589 An Examination of Balance Problems in the Burn Population

**DOI:** 10.1093/jbcr/irae036.223

**Published:** 2024-04-17

**Authors:** Edward Santos, Kaitlyn Chacon, Lauren Shepler, Kara McMullen, Mary D Slavin, Marc van de Rijn, Karen J Kowalske, Colleen M Ryan, Jeffrey C Schneider

**Affiliations:** Spaulding Rehabilitation Hospital, Charlestown, MA; Spaulding Rehabilitation Hospital, Harvard Medical School, Boston, MA; University of Washington, Seattle, WA; Department of Health Law, Policy and Management, Boston University School of Public Health, Boston, MA; Spaulding Rehabilitation Hospital, Boston, MA; UT Southwestern Medical Center, Dallas, Dallas, TX; Massachusetts General Hospital/Shriners Children's, Boston, MA; Spaulding Rehabilitation Hospital, Charlestown, MA; Spaulding Rehabilitation Hospital, Harvard Medical School, Boston, MA; University of Washington, Seattle, WA; Department of Health Law, Policy and Management, Boston University School of Public Health, Boston, MA; Spaulding Rehabilitation Hospital, Boston, MA; UT Southwestern Medical Center, Dallas, Dallas, TX; Massachusetts General Hospital/Shriners Children's, Boston, MA; Spaulding Rehabilitation Hospital, Charlestown, MA; Spaulding Rehabilitation Hospital, Harvard Medical School, Boston, MA; University of Washington, Seattle, WA; Department of Health Law, Policy and Management, Boston University School of Public Health, Boston, MA; Spaulding Rehabilitation Hospital, Boston, MA; UT Southwestern Medical Center, Dallas, Dallas, TX; Massachusetts General Hospital/Shriners Children's, Boston, MA; Spaulding Rehabilitation Hospital, Charlestown, MA; Spaulding Rehabilitation Hospital, Harvard Medical School, Boston, MA; University of Washington, Seattle, WA; Department of Health Law, Policy and Management, Boston University School of Public Health, Boston, MA; Spaulding Rehabilitation Hospital, Boston, MA; UT Southwestern Medical Center, Dallas, Dallas, TX; Massachusetts General Hospital/Shriners Children's, Boston, MA; Spaulding Rehabilitation Hospital, Charlestown, MA; Spaulding Rehabilitation Hospital, Harvard Medical School, Boston, MA; University of Washington, Seattle, WA; Department of Health Law, Policy and Management, Boston University School of Public Health, Boston, MA; Spaulding Rehabilitation Hospital, Boston, MA; UT Southwestern Medical Center, Dallas, Dallas, TX; Massachusetts General Hospital/Shriners Children's, Boston, MA; Spaulding Rehabilitation Hospital, Charlestown, MA; Spaulding Rehabilitation Hospital, Harvard Medical School, Boston, MA; University of Washington, Seattle, WA; Department of Health Law, Policy and Management, Boston University School of Public Health, Boston, MA; Spaulding Rehabilitation Hospital, Boston, MA; UT Southwestern Medical Center, Dallas, Dallas, TX; Massachusetts General Hospital/Shriners Children's, Boston, MA; Spaulding Rehabilitation Hospital, Charlestown, MA; Spaulding Rehabilitation Hospital, Harvard Medical School, Boston, MA; University of Washington, Seattle, WA; Department of Health Law, Policy and Management, Boston University School of Public Health, Boston, MA; Spaulding Rehabilitation Hospital, Boston, MA; UT Southwestern Medical Center, Dallas, Dallas, TX; Massachusetts General Hospital/Shriners Children's, Boston, MA; Spaulding Rehabilitation Hospital, Charlestown, MA; Spaulding Rehabilitation Hospital, Harvard Medical School, Boston, MA; University of Washington, Seattle, WA; Department of Health Law, Policy and Management, Boston University School of Public Health, Boston, MA; Spaulding Rehabilitation Hospital, Boston, MA; UT Southwestern Medical Center, Dallas, Dallas, TX; Massachusetts General Hospital/Shriners Children's, Boston, MA; Spaulding Rehabilitation Hospital, Charlestown, MA; Spaulding Rehabilitation Hospital, Harvard Medical School, Boston, MA; University of Washington, Seattle, WA; Department of Health Law, Policy and Management, Boston University School of Public Health, Boston, MA; Spaulding Rehabilitation Hospital, Boston, MA; UT Southwestern Medical Center, Dallas, Dallas, TX; Massachusetts General Hospital/Shriners Children's, Boston, MA

## Abstract

**Introduction:**

Balance is an important daily functional ability that is dependent on many intrinsic and extrinsic factors; balance impairments can lead to loss of independence, injury, falls, and quality of life limitations. However, balance is not well studied in the burn population. The aim of this study is to describe the frequency of balance impairments and associated long-term factors after burn injury.

**Methods:**

Data from a multicenter longitudinal database from August 2015 to January 2023 was analyzed. Self-reported trouble with balance was collected at discharge and at 6, 12, 24 and 60 months after injury. The population was divided into those with and without balance impairments at 12 months. Demographic and clinical variables were compared between groups. Regression analyses examined the association between demographic and clinical characteristics and self-reported balance impairments at 12 months.

**Results:**

Of the 572 participants that met inclusion criteria, 153 reported balance impairments at 12 months. Balance impairments were most frequently reported at discharge (40.3%) and continued over the five-year follow up periods (26.8 to 36.0%). Those with reported balance impairments were older (53.1±15.7 vs. 45.0±15.9 years) and less likely to be employed (49.0% vs. 74.2%); additionally, they were more likely to have Medicaid or Medicare insurance (22.7% or 31.3% vs 12.7% or 15.1%), inpatient rehabilitation (34.7% vs 21.3%), outpatient physical or occupational therapy at 12 months (41.7% vs 23.7%), vision problems (26.2% vs. 7.0%), leg or feet burns (79.8% vs. 62.1%), numbness in their feet (55.7% vs. 16.8%), and swollen feet or legs (46.3% vs. 14.9%) compared to those without balance impairments (p< 0.001 for all comparisons). Regression analysis demonstrated 4% increased odds of a balance impairment for every increase in year of age (p=0.001). Participants employed at the time of injury had 76% lower odds of reporting a balance impairment at one year (p< 0.05). Additionally, trouble with balance was 279% higher for participants who received outpatient physical or occupational therapy (p=0.005) and 537% higher for those that reported drug misuse (p=0.020).

**Conclusions:**

This study found that balance impairments are commonly reported at discharge and persist for up to 5 years after injury. Results suggest that trouble with balance is more likely to be reported by individuals who are older, unemployed at the time of injury, receiving outpatient therapy services, and misusing drugs.

**Applicability of Research to Practice:**

Balance assessments during inpatient care and follow up appointments may be used to identify persons living with burn injury that may benefit from balance interventions.

